# Breeding Based Remobilization of Tol2 Transposon in *Xenopus tropicalis*

**DOI:** 10.1371/journal.pone.0076807

**Published:** 2013-10-08

**Authors:** Maura A. Lane, Megan Kimber, Mustafa K. Khokha

**Affiliations:** Program in Vertebrate Developmental Biology, Department of Pediatrics and Genetics, Yale University School of Medicine, New Haven, Connecticut, United States of America; Radboud University Nijmegen, Netherlands

## Abstract

*Xenopus* is a powerful model for studying a diverse array of biological processes. However, despite multiple methods for transgenesis, relatively few transgenic reporter lines are available and commonly used. Previous work has demonstrated that transposon based strategies are effective for generating transgenic lines in both invertebrate and vertebrate systems. Here we show that the Tol2 transposon can be remobilized in the genome of *X. tropicalis* and passed through the germline via a simple breeding strategy of crossing transposase expressing and transposon lines. This remobilization system provides another tool to exploit transgenesis and opens new opportunities for gene trap and enhancer trap strategies.

## Introduction

The frog, *Xenopus*, is an ideal vertebrate model for human biology given its evolutionary position among the vertebrates, low cost, and large quantity of embryos amenable to manipulation and molecular techniques [1,2]. To fully exploit *Xenopus*, transgenic reporter lines are necessary to visualize subcellular structures, specific tissues, or dynamic cellular processes [3-6]. Despite the many available transgenesis tools, however, few reporter lines exist in *X. tropicalis*.

An effective scheme for generating reporter lines is enhancer or gene trapping, whereby a transgenic cassette is randomly inserted into the genome and co-opts the expression pattern of a nearby gene [7-14]. Transposon systems are commonly used to introduce these cassettes. One such system is based on Tol2, a member of the hAT (hobo, Ac, and Tam) DNA transposon family, originally isolated as an autonomous element from the Medaka fish *Orzyias latipes* [15,16], and developed as a non-autonomous system [17] for use in transgenesis. This “cut and paste” transposon inserts canonically at a random heterogenic sequence, often at multiple loci, and creates a signature eight base pair (bp) target site duplication (TSD) [18]. Tol2 has applications for *Xenopus* transgenesis [19-21], has demonstrated “local hopping” [22-25], and has been shown to favor insertion into transcriptional regions of genes [24-26], all properties which make it highly attractive as a gene and enhancer trapping tool in frog.

Sleeping Beauty, in the Tc1/mariner family of transposons, is another transposon system commonly used to insert transgenic cassettes into the genome. It differs from Tol2 in a number of characteristics affecting integration or remobilization of transposons. For example, Sleeping Beauty targets a random TA sequence for integration, shows a preference to integrate a low copy number concatomer in intergenic regions, and has a very high tendency for local hopping [26,27].

Once inserted into the genome, transposons can be remobilized by transient transposase re-expression, resulting in novel integrations and further opportunities for enhancer trapping. In Drosophila and Zebrafish, transposon remobilization and screening of transgenic lines is common and effective [23,24,28-30]. In *X. tropicalis*, remobilization of transposons has been demonstrated using two methods: 1) microinjection of transposase mRNA (Tol2) [22] and 2) breeding transposon lines to transposase lines (Sleeping Beauty) [27].

In the microinjection method [22], *in vitro* transcribed Tol2 mRNA injected in embryos bearing the Tol2 transposon resulted in a very high remobilization efficiency (18/18 injected embryos showed evidence of remobilization) [22]. The micro-injection method is labor intensive however, as transposase mRNA is prepared and embryo injections performed each time remobilization is desired.

In the breeding based remobilization strategy employing Sleeping Beauty [27], a transgenic animal with the Sleeping Beauty transposon was crossed with one from another line harboring a corresponding transposase expressing construct. Exposure to the transposase resulted in remobilization and re-integration of substrate transposons. This breeding based strategy has thus far proven less efficient than mRNA injection in producing remobilization and reintegration in *Xenopus*, less than 1% on average [27]. While less efficient than mRNA injection, the breeding based strategy is less labor intensive, only requiring setting up a mating, then collecting and analyzing embryos.

As with injection based transposon transgenesis methods, it is advantageous to have multiple types of transposon systems available for use with breeding based remobilization strategies. Thus, a transposon system such as Sleeping Beauty may be useful in gene therapy applications, where non-genic insertions are desirable, while one such as Tol2 may be useful for enhancer trap screens, where genic insertions are valuable [25]. Multiple transposon systems used together could also harness the advantages of each for different applications such as enhancer or gene trapping.

Currently, *Xenopus* lacks the remarkable repertoire of transgenic lines available for other systems. A breeding based remobilization strategy using Tol2 would provide a new platform to create currently lacking reporter lines by enhancer or gene trapping in *Xenopus*. Here we demonstrate this strategy for the first time by crossing Tol2 transposon and transposase lines in *X. tropicalis*. We identify readily apparent remobilization in an average of 1.3% of embryos, and we are able to successfully map new integrations in 67% of these embryos.

## Materials and Methods

### Ethics Statement

The animal protocol was approved by the Yale University Institutional Animal Care and Use Committee (Animal Use Protocol Number: 2012-11035).

### Husbandry

*X. tropicalis* were housed and cared for in our aquatics facility according to established protocols approved by the Yale University Institutional Animal Care and Use Committee.

### Plasmids and generation of transgenic lines

#### Tol2 transposon *X. tropicalis* animals

**Figure 1:**
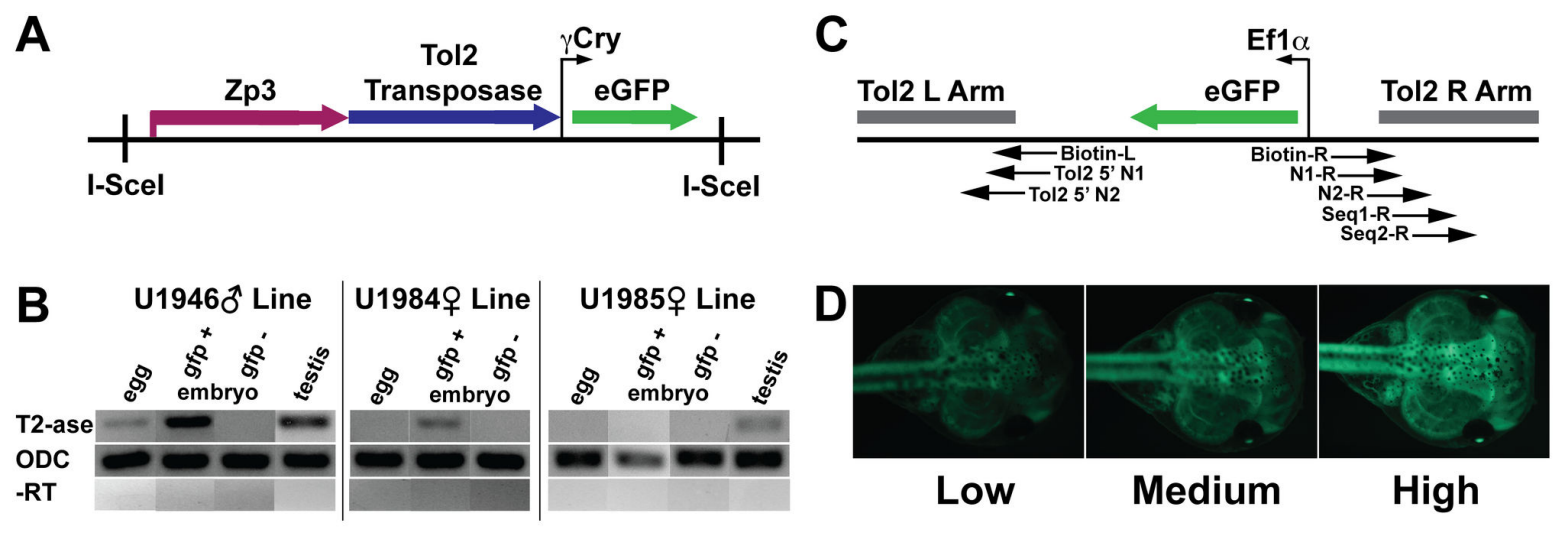
Transgenic lines. A. Diagram of ZP3T2γGMN construct used to produce transgenic frogs expressing Tol2 transposase. Not to scale. I-SceI: Meganuclease site necessary for transgenesis. Zebrafish zona pellucida glycoprotein 3 (zp3) promoter: drives egg specific expression of Tol2 transposase. Gamma crystallin promoter (γCry): drives expression of eGFP in lens of the eye as a reporter for transgene insertion. B. RT-PCR for transposase expression in transgenic ZP3T2γGMN F1 offspring arising from outcrosses of transposase transgene injected animals U1946♂, U1984♀ and U1985♀. Pools of 10 egg, stage 30 gfp+ and gfp- embryos were tested. In the U1946♂ and U1985♀ lines, one testis from adult male frogs was also tested. OCD primers were used as positive controls (- RT). reactions using GFP+ embryos, and water (data not shown) were also used as negative controls. C. Diagram of Ef1αGFPTol2 construct. Tol2 left (L) and right (R) arms, EF1α enhancer driving eGFP transgene. Arrows: indicate specified LM-PCR and sequencing primer binding sites on transposon arms. D. Ubiquitous GFP+ phenotypes. Low, Medium, and High Intensity phenotypes were seen in F3 and F4 embryos.

**Figure 2:**
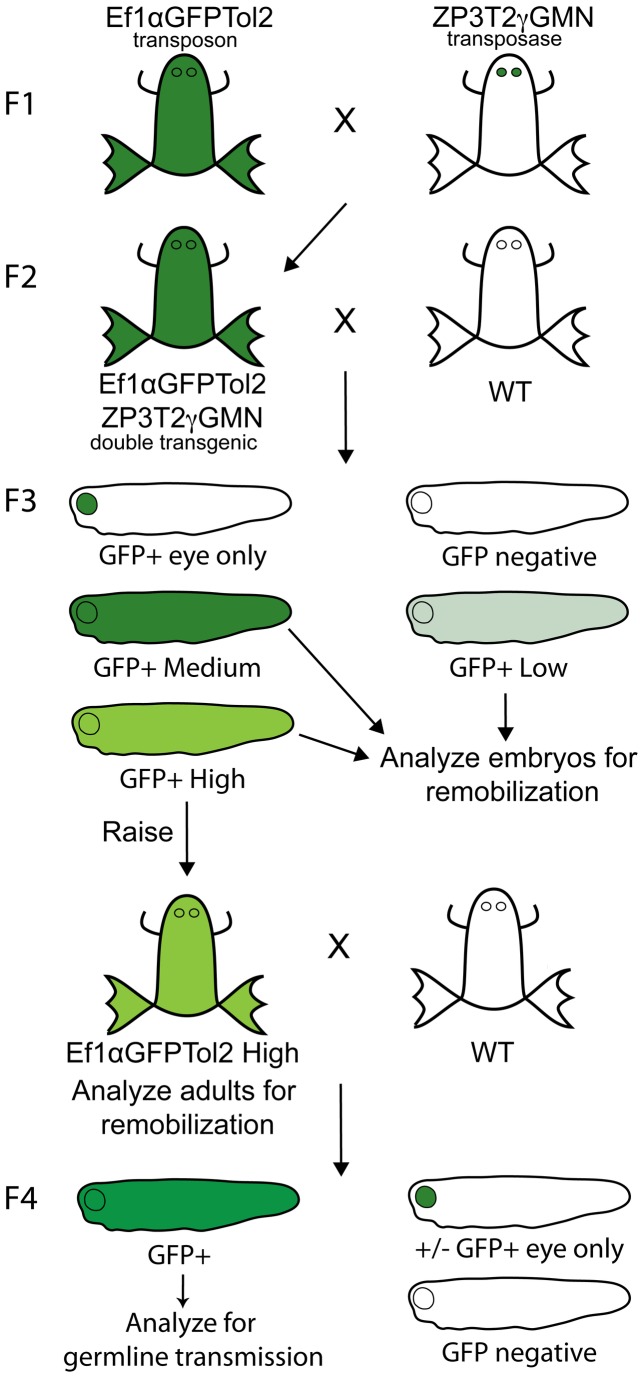
Mating scheme for generation and analysis of Ef1αGFPTol2 remobilized transposons. The diagram is a schematic of the crosses to test transposon mobilization in each of three different lines. We crossed three F1 heterozygous ZP3T2γMN transposase frogs (one from each injected tranposase animal U1946♂, U1984♀ and U1985♀) with three F1 homozygous Ef1αGFPTol2 frogs to generate three F2 double transgenic (Ef1αGFPTol2/ZP3T2γGMN) offspring clutches. Double transgenic animals from each clutch were outcrossed, and F3 ubiquitous GFP+ embryos of each phenotype observed were collected and tested for remobilization. High intensity F3 embryos, found only in the U1984♀ line, were raised to adulthood, tested for remobilization, and outcrossed to test for germline transmission.

We obtained heterozygous Tol2 transposon animals derived from the “10M” female founder, who harbors a single Tol 2 insertion mapping to scaffold 8 of the JGI Genome v. 7.1 (animals kindly provided by Paul Mead) [20]. This transposon, which we call Ef1αGFPTol2, contains an EF1α enhancer which drives eGFP ubiquitously in the embryo (Figure 1C). We confirmed the insertion locus in our heterozygous animals by linker-mediated PCR (LM-PCR, see below). This insertion site became our substrate for testing transposon remobilization. We bred the heterozygous animals to homozygosity, and selected three homozygous animals as F1 founders to cross with our F1 transposase animals (described below and Figure 2 and Figure S1).

#### Generation of ZP3T2γGMN transposase *X. tropicalis* lines

We cloned the zp3 (zona pellucida 3) promoter (kindly provided by Z. Gong) [31] adjacent to the Tol2 transposase (T2; kindly provided by K. Kawakami) [32]. Adjacent to this cassette, we included a γCrystallin-eGFP cassette in order to follow transposase inheritance in our frogs (γG; kindly provided by Rob Grainger) [3]. This ZP3T2γG cassette was then subcloned into a meganuclease vector with flanking I-SceI sites (Figure 1A).

We created three ZP3T2γGMN transposase transgenic lines using the meganuclease method [33], which tends to result in a concatomer of multiple transgene integrations at a single locus. Three injected ZP3T2γGMN animals (U1946**♂**, U1984**♀**, and U1985**♀**, Figure S1) showing GFP expression in the eye (GFP+^eye^) at stages 38-40 [34] were raised and outcrossed with WT frogs to create three F1 clutches with GFP+ eye expression (C882, C913, C914 respectively, Figure S1). In order to identify F1 animals bearing a single insertion locus of the transposase transgene, a subset of F1 animals from each clutch were raised and outcrossed to wild-type animals (Figure S1). We selected three F1 animals with transgene transmission rates consistent with a single insertion (i.e 50% of F2 animals were GFP+^eye^) to cross with our Ef1αGFPTol2 transposon animals (Figure 2 and Figure S1). Anticipating that different expression levels of transposase may lead to different transposon remobilization efficiencies, we did not characterize the insertion locus for copy number by Southern blot, but instead chose to analyze expression levels by RT-PCR, and then empirically test for remobilization rates. For simplicity, we call the three transposase lines the U1946**♂**, U1984**♀**, and U1985**♀** lines, referring to the original injected animal giving rise to each line (Figure S1).

#### Generation of double transgenic Ef1αGFPTol2 x ZP3T2γGMN frogs

One male and two female F1 heterozygous GFP+^eye^ ZP3T2γGMN animals were generated as described above. We crossed these F1 animals with our homozygous F1 Ef1αGFPTol2 animals to produce three double transgenic F2 clutches (C1030, C1039, C1040, Figure S1). A subset of each of these clutches harbored both one copy of Ef1αGFPTol2 transposon and ZP3T2γGMN Tol2 transposase constructs (Figure 2). Because the ubiquitous GFP expression of our Ef1αGFPTol2 transposon line masked the GFP eye marker of our ZP3T2γGMN transposase line, any F2 animals that did not produce eye only expression in F3 embryos in an outcross were euthanized. Figure S1 contains detailed information regarding the generation of the three double transgenic lines. Lines are available upon request. If we receive many requests, we will submit the lines to the National *Xenopus* Resource at the Marine Biological Laboratroy in Woods Hole, MA.

### RT-PCR analysis of ZP3T2γGMN transposase expression

We isolated total RNA from eggs and stage 30 embryos from outcross matings of GFP+^eye^ F1 ZP3T2γGMN female frogs in each transposase line. We first primed these females with 10 U Human Chorionic Gonadotropin (HCG), and boosted with 200 U HCG approximately 12 hours later. We collected pools of 10 eggs for each sample, then collected pools of 10 stage 30 embryos post-fertilization. We collected testes from GFP+^eye^ F1 adult male frogs in U1946**♂** and U1985**♀** lines, and used one testis for each sample. We isolated total RNA from all samples using a standard phenol/chloroform extraction protocol.

We performed first strand cDNA synthesis using Super-Script III First Strand Synthesis System (Invitrogen, Cat. # 18080051) according to manufacturer’s protocols. We used 1 µg of RNA per RT reaction. Primers for the ZP3T2γGMN construct (tol2_transposase_F and tol2_transposase_R, Table S1) amplified a 265 bp fragment (Figure 1B). ODC primers (Table S1) were used as a loading control. GFP- embryos, GFP+(-RT) (Figure 1B), and water (data not shown) reactions were used as negative controls.

### GFP Expression Analysis

We detected GFP expression using a Zeiss SteREO Lumar.V12 fluorescent microscope and recorded images with a AxioCamHR3 digital camera in conjunction with AxioVision AxioVS V4.8.1.0 imaging software.

Embryos from three F2 double transgenic clutches, C1030, C1039 and C1040, arising from the three transposase injected animals U1946**♂**, U1984**♀** and U1985**♀** respectively (Figure S1), were screened for overall number of GFP+ and GFP- embryos, as well as for differences in fluorescent intensity, spatial and temporal GFP expression (Table 1). Examination of the tadpoles did not reveal any changes in spatial or temporal expression, but we did notice that the intensity of the ubiquitous GFP signal varied in a small percentage of tadpoles. We then characterized tadpoles as either High Intensity, Medium Intensity, or Low Intensity, where the majority of tadpoles were of Medium Intensity, similar to the unmanipulated Ef1αGFPTol2 baseline expression. As described in Results (below), we focused our attention on the High Intensity tadpoles. We counted the number of GFP+ tadpoles, identified those that were High Intensity, and reported cumulative totals (Table 1).

**Table 1:** GFP segregation in tadpoles from double transgenic outcross.

ZP3T2γGMN Line	F2 Tol2/ZP3T2γGMN	F3 GFP+ to Total Embryos	F3 High GFP+ to Total GFP+ Embryos
U1946 **♂**	C1030	1125/2169 (51.9%)*	0/2822 (0%)
U1985 **♀**	C1040	404/817 (49.4%)	0/404 (0%)
U1984 **♀**	C1039 U2521**♂**	657/1353 (48.6%)*	11/957 (1.15%)
	C1039 U2522**♂**	2959/6038 (49.0%)*	78/5576 (1.40%)
	C1039 U2644**♀**	386/554 (69.7%)	7/386 (1.81%)
	C1039 U2645**♀**	492/903 (54.5%)	7/492 (1.42%)
	C1039 U2646**♀**	921/1763 (52.2%)	6/921 (0.65%)
	C1039 U2647**♀**	928/1713 (54.2%)	19/928 (2.04%)

Column 1: ZP3T2γGMN injected animals, U1946 ♂ U1985 ♀ and U1984 ♀ were used to create three double transgenic F2 lines (C1030, C1040, C1039 respectively) as described in Methods and Figure S1. Column 2: We scored these three F2 double transgenic clutches for GFP phenotypes. C: Clutch identification number. U: Unique animal identification number. Column 3: Ratio of total ubiquitous GFP+ embryos to total number of embryos from outcross of double transgenic frogs. Column 4: Ratio of High Intensity embryos to total number of GFP+ embryos from outcross of double transgenic frogs. High Intensity embryos were only found in C1039 animals.

For clutches C1030 and C1040, the results in Columns 3 and 4 are the cumulative totals from outcrosses of a number of animals (C1030: 1 ♂ and 8 ♀ C1040: 1 ♂ and 5 ♀). For clutch C1039, results in Columns 3 and 4 are the cumulative totals from multiple outcrosses of each unique animal except U2644 ♀ where results are from a single outcross only. In the three cases indicated by * we determined the ratio of GFP+ embryos to total number of embryos from a subset of all the embryos scored. We then identified GFP+ embryos from all embryos collected. From this larger set of GFP+ embryos, we identified High Intensity embryos and calculated the ratio of High Intensity GFP+ embryos to total GFP+ embryos (Column 4). As a result, the denominator in the fourth column is larger than the numerator in the third column.

### Transposon Integration Site Analysis

#### Extension Primer Tag Selection Linker Mediated PCR (EPTS LM-PCR)

We performed integration site analysis on left and right Tol2 transposon arms using an EPTS LM-PCR protocol, as previously described [35]. Briefly, a frequently cutting endonuclease is used to cut genomic DNA close to the transposon arm. A biotinylated primer complementary to the transposon arm is hybridized to the fragments, incubated with septavidin beads, and isolated after multiple washes on a capture magnet. Oligos are ligated onto the genomic end and then the transposon-genomic junction is amplified with primers specific to the transposon arm and ligated oligo. We modified the published protocol by using the restriction enzymes MspI and NlaIII in combination with different primers designed on the Tol2 right arm (Figure 1C, Table S1) to amplify the right arm transposon-genomic junction. LM-PCR products were either sequenced directly (using primers Tol2 5’ N1 and N1-R or Seq1-R, Table S1), or cloned into TOPO-TA vector (Invitrogen, Carlsbad, CA, USA) and then sequenced using standard M13 forward and reverse primers in the vector.

We performed EPTS LM-PCR integration site analysis using DNA from individual stage 40 whole F3 and F4 tadpoles, and using DNA from toe clip tissue of adult F3 High Intensity animals (Figure 2) prepared with the Qiagen DNeasy Blood and Tissue kit “Purification of Total DNA from Animal Tissues” protocol. We did not test any somatic tissue for evidence of somatic remobilization.

#### Genomic PCR (gPCR)

If we could identify the insertion site of only one transposon arm by LM-PCR, we attempted to amplify the other transposon arm based on its presumed genomic locus ascertained from the successfully cloned arm. We used genomic (Table S1) and transposon (Tol2 5’ N2 and Seq1-R, Table S1) primer combinations to amplify sequence at both left and right transposon arm junctions. We designed genomic primers based on sequence flanking the left and right transposon junction of each putative insertion where possible. In some cases, repetitive sequence made designing primers problematic.

We used the following PCR conditions: denaturation at 94°C for 2 min, followed by cycles of 94°C for 15s, 30s at annealing temp, followed by an elongation step at 72°C. For expected PCR products less than 500 bp, the elongation time was 30s, for 500-1000 bp 60s, and for >1000 bp 90s. Initially we used an annealing temperature of 55°C, and then tried 53°C or 52°C if there was no amplification.

We examined PCR amplification products by gel electrophoresis and then isolated and purified PCR products using the Qiagen Gel Purification kit. We cloned purified PCR products into TOPO-TA before sequencing. Sequences were blasted against the Joint Genome v. 7.1 (http://www.xenbase.org). In some cases, we were unable to clone the integration site due to failure to amplify the integration site or repetitive sequence that made identifying a unique site impossible.

## Results

### Test for Transposase Expression

We first tested F1 ZP3T2γGMN transgenic frogs from each line for transposase expression by RT-PCR (Figure 1B). In the U1946**♂** line, expression was detected in eggs and GFP+^eye^ embryos from the female frogs and in the testis of adult male frogs. We did not detect expression in eggs from female frogs in the U1984**♀** line, but expression was present in GFP+^eye^ embryos. In the U1985**♀** line, we did not detect expression in the egg or embryo of female frogs, but found expression in testis of adult male frogs (Figure 1B).

### Screen Double Transgenic Offspring for GFP Expression

To ensure the GFP+ and GFP- embryos were segregating at expected 1:1 frequencies, we outcrossed double transgenic animals from each transposase line as described in Methods, and scored for overall number of GFP+ embryos (Table 1, Column 3). Because the ubiquitous GFP expression of our Ef1αGFPTol2 transposon line masked the GFP eye marker of our ZP3T2γGMN transposase line, any F2 animals that did not produce eye only expression in an outcross were euthanized. In cases where the transposase marker was present, overall ubiquitous GFP+ phenotype frequencies reflected simple Mendelian inheritance of the Ef1αGFPTol2 transposon in the F3 generation in most cases. In the case of U2644**♀** (from C1039 in the U1984**♀** line), the higher than expected percentage of GFP+ embryos (69.7%) is likely due to the presence of bright maternal GFP.

We next scored each outcross for GFP expression differences in GFP+ embryos. We identified three ubiquitous GFP+ fluorescent intensities, categorized as High, Medium, and Low Intensity (Figure 1D). High Intensity embryos were very bright and easily identified visually. Subtle differences in intensity were found among the High Intensity embryos, but were all categorized as High Intensity for the purposes of this study. The intensity of the Medium Intensity embryos was identical to that of their double transgenic parents. Low Intensity embryos showed the least fluorescence. We did not note any change in the GFP+ expression intensity or pattern in the embryos over the observed time period of fertilization to stage 40.

The U1984**♀** line is the only line in which we found the High Intensity phenotype. We screened 2 male and 4 female frogs in this line (Table 1, Column 2). All three phenotypes were present in embryos from each mating, with the majority being Medium Intensity, and a small percentage having Low (avg. 2.0%) and High Intensity expression (0.65-2.04%) (Table 1, Column 4).

We screened 1 male and 8 female frogs in the U1946**♂** line. The majority of embryos were Medium Intensity, and a small percent were Low Intensity (data not shown). In the U1985**♀** line, we saw only Medium Intensity expression in screening 1 male and 5 female double transgenic frogs. We did not find evidence of High Intensity embryos in the U1946**♂** or U1985**♀** lines (Table 1, Column 4).

### Test for Remobilization

Embryos of each intensity (Low, Medium, High) seen in the F3 generation were tested for transposon remobilization by LMPCR +/- gPCR (Figure 2). We found evidence of remobilization only in High Intensity embryos, observed solely in the U1984**♀** line (Figure S1). We found apparent remobilization in an average of 1.3% of embryos the U1984**♀** line (Table 1, Column 4), and we were able to successfully map new integrations in 67% of these embryos (Table 2). We tested 107 embryos collected from 2 male and 4 female double transgenic frogs from the U1984**♀** line. Remobilized transposons were found in the offspring of both male and female frogs. We identified 45 novel PCR products in 42 High Intensity embryos. All successfully mapped High Intensity embryos showed both the original transposon and a new integration. 29 new integrations were mapped on both transposon arms, including confirmation of the TSD (Table 2). One integration was successfully mapped on one arm only; the second arm was not mapped due to repetitive sequence (Table 2, U2646F HI#2). Lack of quality sequence or repetitive sequence on both arms prevented mapping to any loci for 12 samples. In three cases, we were unable to obtain any PCR product. No new PCR products were seen in the 23/23 Low Intensity and 42/42 Medium Intensity embryos tested in the U1984**♀** line.

**Table 2:** Integration site analysis.

Parent	F3	Genomic: LA Tol2 sequence	Genomic: RA Tol2 sequence	JGI (v.7.1)	Flanking gene	kbp to gene
F1 Tol2		..ACCTGGAAC**CCCGAAAT**cagaggtgta..	..tacacctctg**CCCGAAAT**CCGCAGACT..	8:87633873	LOC1001251672	intron 1
F2 U2521**♂**	HI #1	.. TAAGATATA**ATTACCCT**cagaggtgta..	.. tacacctctg**ATTACCCT**TATTGGATG..	2:132651708	Xetro.B02176	126.7
	HI #2	..AGCACGACC**CACACATC**cagaggtgta..	..tacacctctg**CACACATC**ATTGTCAAT..	8:81162239	slc38a6	45.4
	HI #3	..CCTTGCAAA**CATCTGTC**cagaggtgta..	..tacacctctg**CATCTGTC**CATTGGGAA..	1:124132951	LOC4947062	4.4
F2 U2522**♂**	HI #4	..GTCATTTGC**CTTATACT**cagaggtgta..	..tacacctctg**CTTATACT**GAAGTTTGC..	1:14294794	trappc13	intron 7
	HI #5	..GTGTGCGAC**GTCAGCAC**cagaggtgta..	..tacacctctg**GTCAGCAC**GCACAGGGC..	505:45386	Xetro.K02900	4.1
	HI #7	..TGTCTATTG**TCTTTAGA**cagaggtgta..	..tacacctctg**TCTTTAGA**TCACCTAAT..	8:83640859	lgals3	0.3
	HI #12a	..TTAAAATCC**CTTATTAG**cagaggtgta..	..tacacctctg**CTTATTAG**GGCCACACT..	8:30665922	utp14a	1.3
	HI #13	..AGAGGGTAT**AGGAGTAG**cagaggtgta..	..tacacctctg**AGGAGTAG**GTTAAGGAT..	8:84762850	ubr1	1.8
	HI #14	..TGGGCATTG**GTTTCAGT**cagaggtgta..	..tacacctctg**GTTTCAGT**CATAGGGCT..	8:48893707	gng8	2.9
	HI #16	..CCCTTCTCC**ATTAAGGA**cagaggtgta..	..tacacctctg**ATTAAGGA**GATCCCCCC..	3:47246149	anapc13.2	16.9
	HI #18	..GCCTATTGA**CACACAGC**cagaggtgta..	..tacacctctg**CACACAGC**ACTGTGCAA..	6:9467577	has2	18.7
	HI #19	..TTGGCCTTG**GTGATTAC**cagaggtgta..	..tacacctctg**GTGATTAC**TGATAAGCA..	8 87693428	dpf3	29.3
	HI #20	..GAAAGATCC**TTTAAATT**cagaggtgta..	..tacacctctg**TTTAAATT**AACTTTTAG..	8:84932662	stard9	intron 10
	HI #21	..TATTTAGCA**CGCAAAAG**cagaggtgta..	..tacacctctg**CGCAAAAG**TTATAGTGA..	8:87933146	rgs6	30.5
	HI #22	..CTCGTATCC**ATTATAAT**cagaggtgta..	..tacacctctg**ATTATAAT**ATATGCTGT..	8:88268806	pcnx	intron 23
	HI #23	..ATTAATCTA**TTGATGGC**cagaggtgta..	..tacacctctg**TTGATGGC**CTAATTCTG..	8:86090368	cfl2	intron 1
	HI #24	..AAATCGATA**CTCAGAAG**cagaggtgta..	..tacacctctg**CTCAGAAG**TCAGCACTG..	1:86927815	nfil3	5' UTR intron
	HI #25a	..ACAGTGCAA**CATATACT**cagaggtgta..	..tacacctctg**CATATACT**TATATTTAA..	184:11129	slc12a4	51.8
	HI #25b	..TTTAGAGAT**ATAATGAT**cagaggtgta..	..tacacctctg**ATAATGAT**ATTTTTCAA..	4:100939936	klf17	24.7
	HI #26	..AAACACCTG**GGTACCGC**cagaggtgta..	..tacacctctg**GGTACCGC**AGGTCTAAA..	9:17814879	sp5	2.2
F2 U2644**♀**	HI #3	..AGTTAAACC**ACCTTAAG**cagaggtgta..	..tacacctctg**ACCTTAAG**TGCGACTTG..	8:88132604	sipa1l1	5' UTR intron
	HI #5	..ACAGTCGCC**ATCTTGCT**cagaggtgta..	..tacacctctg**ATCTTGCT**ACAATGTAA..	8:87633302	Xetro. H01750.1	16.2
	HI #6	..CCTATTTGT**AACCCCTG**cagaggtgta..	..tacacctctg**AACCCCTG**GAACATTTT..	8:76909153	klc1	10.8
F2 U2645**♀**	HI #2	..ACATACACA**CACACAGC**cagaggtgta..	..tacacctctg**CACACAGC**ATCATGTGA..	8:49467657	mark4	intron 1
	HI #3	..GCTACGTGT**ATGGCCAC**cagaggtgta..	..tacacctctg**ATGGCCAC**CTTAAATCT..	8:82067778	daam1	5' UTR intron
	HI #4	..AAGTGTGGT**CACCTTGG**cagaggtgta..	..tacacctctg**CACCTTGG**CTGCAGTTC..	8:53558200	Xetro.H01082	6.8
	HI #5	..TTTGTATCA**ATACAACC**cagaggtgta..	..tacacctctg**ATACAACC**ATATAAAGG..	8c:4355834	Xetro.K05083	9.0
	HI #6b	..AATAGTTGA**GGAGCAAC**cagaggtgta..	..tacacctctg**GGAGCAAC**ACAAGCATG..	7:99952578	dpb	intron 2
F2 U2646**♀**	HI #21	..GGGGTATTA**GTGGCAAA**cagaggtgta..		8:83208318	tmem260	intron 3
F2 U2647**♀**	HI #1	..CTGTCATAC**TGAAACAG**cagaggtgta..	..tacacctctg**TGAAACAG**TGACTAAGT..	1:165003122	Xetro.A02812	137.2

Row 1: F1 Tol2 (Ef1αGFPTol2) is the original donor transposon insertion site. Novel amplicons from High Intensity F3 offspring from six F2 double transgenic founders (Ef1αGFPTol2/ZP3T2γGMN) are listed in subsequent rows. Column 1: F2 Double transgenic founder animal. Column 2: Novel amplicons from F3 HI expressing offspring. Columns 3 and 4: Left and right flanking genomic sequence at transposon insertion site are in uppercase. Genomic Target Site Duplication (TSD) sequence is in bold. Transposon sequence is in lowercase italics. Column 5: Genomic insertion site (JGI Genome v. 7.1), with chromosome number: base pair position. Column 6: Most proximal flanking gene. Column 7: Distance to most proximal flanking gene (kbp). ^1.^One arm successfully maps, but sequence flanking other transposon arm is poor quality or not unique. ^2.^Predicted gene.

**Figure 3:**
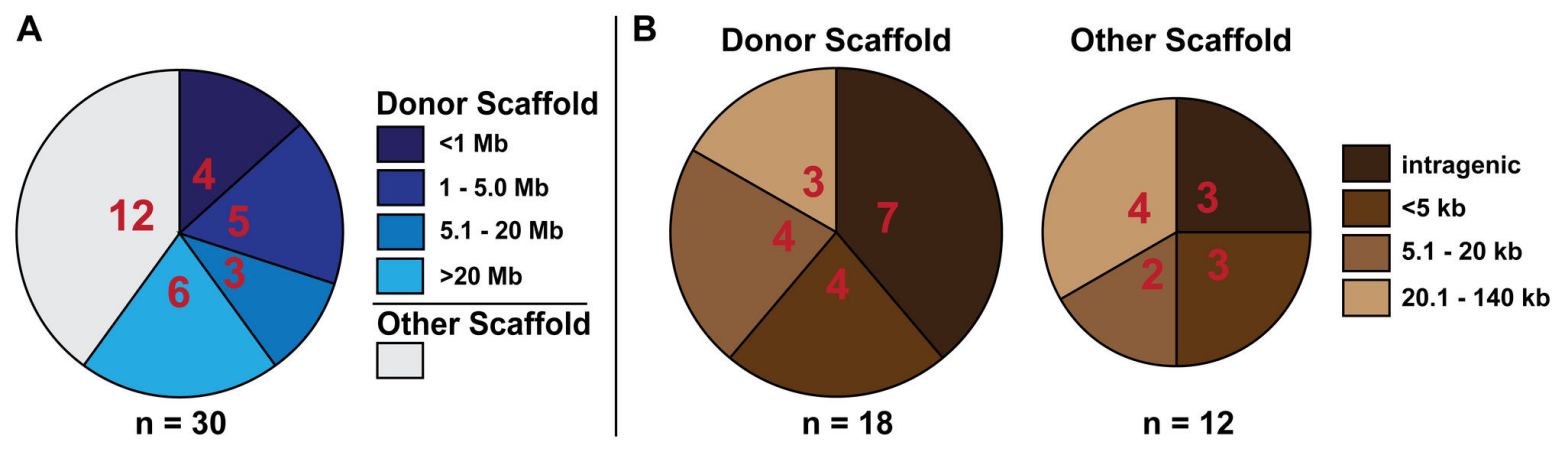
Remobilization Data. A. Number of remobilizations mapped to donor scaffold 8 compared to those mapped to other scaffolds. Proximity (Mb) of remobilized transposons on scaffold 8 to the donor locus is also shown. B. Number of intragenic versus intergenic integrations for remobilizations on both donor and other scaffolds, including proximity (kb) of intergenic integrations to the nearest flanking gene. The size of the pie charts indicates the relative number of remobilizations on donor versus other scaffolds. Number of samples in each category are shown within the pie slices for both A and B, and total n numbers are indicated beneath each graph.

Remobilizations found relatively close to the donor insertion, or on the donor chromosome, are frequently referred to as “local hops” [23,24,27]. 60% (18) of confirmed remobilizations in our study were on the same scaffold as the parental insertion, and ranged in distance from 571 bp to 83.3 Mb to the donor insertion, with 50% being within 5 Mb of the donor insertion, and 22% within 1 Mb of the donor (Figure 3A). The remaining 40% (12) of remobilizations were found on non-donor scaffolds (Figure 3A).

We found that 61% (11) of remobilizations on the donor scaffold were intragenic or less than 5 kb from a gene (Figure 3B, Table 2), a distance that may encompass a gene’s regulatory region [24]. The high percentage of these new insertions in close proximity to a gene may be due to the relatively gene rich area in which the donor transposon is located. 50% (6) of remobilizations on non-donor scaffolds were also in or within 5 kb of a gene (Figure 3B, Table 2). 90% (9) of intragenic remobilizations were in introns in the 5’ region of the gene. Though none of the intragenic remobilizations are in exons, two were less than 500 bp away from an exon (Table 2, U2522M HI#22, U2645F HI#2).

We did not find evidence of remobilized transposons in any embryos from the U1946**♂** or U1985**♀** lines. We tested 14 Low Intensity and 68 Medium Intensity embryos from 1 male and 8 female double transgenic frogs in the U1946**♂** line. No evidence of reintegration was found in any of the embryos, and this line was not tested further (Figure S1). In the U1985**♀** line, we tested 46 Medium Intensity embryos collected from 1 male and 5 female double transgenic frogs, and found no evidence of remobilization (Figure S1). As these animals also showed little transposase expression (Figure 1B), this line was subsequently culled.

### Germline Transmission

To confirm stable germline transmission of the reintegrated transposons, F3 High Intensity embryos from F2 double transgenic male U2522**♂** in the U1984**♀** transposase line were raised to adulthood (Figure 2). Genomic DNA was collected from the adult F3 frogs, and tested for transposon remobilization by LM-PCR (Table 2, samples U2522M HI# 12a -26). We selected three of these F3 animals (U2625**♂**, U2634**♀**, U2635**♂**, Figure S1), each harboring the donor transposon and one remobilized transposon, to outcross (Table 3, Column 2). Two of these frogs, U2625**♂** and U2634**♀**, had remobilized transposons on non-donor chromosomes (Table 2, U2522M HI#24 and HI#18 respectively, and Table 3, Column 3). The third animal, U2635**♂**, harbored the donor insertion and a local remobilization on scaffold 8, 635 kb from the donor insertion (Table 2, U2522M HI#22 and Table 3, Column 3).

**Table 3:** Germline Transmission of Remobilized Transposons in U1984♀ line.

F2 Double Transgenic	F3 High GFP+	F3 Genotype	F4 GFP+ Intensity	F4 GFP+/total embryos	F4 Sequenced/Confirmed	F4 Genotype
C1039 U2522 **♂**	U2625**♂**	S8, S1	Low	50/154 (32%)	3/3	S8
			Medium	35/154 (23%)	2/2	S1
			High	41/154 (27%)	3/3	S8 and S1
	U2634**♀**	S8, S6	Medium	67/168 (40%)	6/6*	S8 or S6
			High	62/168 (37%)	3/3	S8 and S6
	U2635**♂**	S8, S8^☨^	High	116/241 (48%)	2/2	S8 and S8^☨^

Column 1: We tested germline transmission in F2 double transgenic (Ef1αGFPTol2/ZP3T2γGMN) U2522**♂** (see Figure S1), whose F3 embryos showed remobilization (Table 2). We raised F3 GFP+ High Intensity embryos from U2522**♂**, and confirmed remobilization in these animals by LM-PCR. Columns 2 and 3: We outcrossed three of these F3 animals, U2625**♂**, U2634**♀**, and U2635**♂**, each harboring the original donor transposon, as well a new remobilized insertion. Columns 4 and 5: We scored F4 embryos from each outcross for different GFP+ intensities, as well as ratio of those intensities to overall number of embryos. Column 6: We sequenced the transposon insertion sites of a number of embryos of each intensity to confirm stable germline transmission of the insertions found in the F3 parent. Column 7: In two cases, U2625**♂** and U2634**♀**, transposons segregated independently following simple Mendelian inheritance. In the final case, U2635**♂**, transposons appeared linked and were in fact located on the identical chromosome. C: Unique clutch identification number. U: Unique animal identification number. * 4/6 embryos of the Medium GFP+ phenotype in the U2634**♀**cross were confirmed to have the original insertion on S8, and 2/6 were confirmed to have the insertion on scaffold 6. S8 : Original donor insertion on scaffold 8. S8^☨^ : Remobilized insertion on donor scaffold 8.

We scored F4 embryos from outcrosses of these F3 High Intensity animals for fluorescent expression level and for numbers of embryos of each expression level present (Table 3, Columns 4 and 5). We found embryos from U2625**♂** showed three expression levels, High, Medium and Low Intensity, while embryos from U2634**♀** had two expression levels, Medium and High Intensity. Embryos from both U2625**♂** and U2634**♀** had different intensities present in percentages consistent with two independently assorting transposon insertions, as expected since the remobilized transposon was on a non-donor chromosome (Table 3, Columns 4 and 5). We observed only High Intensity F4 embryos from U2635**♂**, whose remobilized insertion was found to be 635 kb from the original insertion on scaffold 8. In the case of transposon insertions linked by close proximity, all of the offspring would inherit both insertions, and would be expected to have the same phenotype (Table 3, Columns 4 and 5).

We performed EPTS LM-PCR on a number of embryos of each expression level in the F3 outcrosses described above (Table 3, Columns 4, 6 and 7). This confirmed the correlation of the Low and Medium Intensity expression to embryos with single insertions, and the High Intensity expression to embryos inheriting both insertions. Of note, in the case of U2634**♀**, embryos inheriting only one transposon insertion (the original insertion on scaffold 8 or the remobilized transposon on scaffold 6), each showed an identical Medium Intensity fluorescence. We tested 6 of these embryos by LM-PCR, and found that 4 embryos harbored the original insertion, and 2 had inherited the new insertion on scaffold 6.

## Discussion

In this study, we show a substrate Tol2 transposon can be remobilized by crossing to transposase expressing animals. We believe this strategy is a viable and efficient method of screening for new insertions when those embryos showing increased fluorescence (High Intensity) are selected for testing. This phenotype was easy to identify in a dish of embryos, and we were able to successfully map reintegrations in the majority of these embryos. Given the remarkable fecundity of *X. tropicalis*, even a relatively low remobilization rate can result in a substantial number of remobilized transposons. All of the successfully mapped High Intensity F3 embryos showed both the original transposon and the new integration. A similar result has been found in other studies of Tol2 remobilization, and has been postulated to be a result of transposon duplication in the S-phase of mitosis [22,24].

While we did not find any evidence of reintegration in Low and Medium Intensity F3 embryos, it may be occurring rarely enough to be difficult to detect without screening very large numbers of embryos. Thus, it is possible remobilization rates are higher than we have found, but random selection of embryos has proven to be an inefficient method of finding reintegrations. Overall, our apparent remobilization rate using the breeding based method was relatively low, a range of 0.65-2.04% among different F2 animals (Table 2), an average of 1.3%. A breeding based remobilization strategy using the Sleeping Beauty transposon system [27] in *Xenopus* showed similar efficiency to our study in producing reintegration in offspring, less than 1% on average. An injection based Tol2 remobilization strategy [22] resulted in a very high remobilization efficiency, where 18/18 injected embryos analyzed showed evidence of at least one new integration. These results are similar to those found in Tol2 remobilization studies in zebrafish, where remobilization by injection resulted in relatively high apparent germline remobilization rates (8.3-100% among different animals in one study [23] and 38% and 48% from two screens in another [24]). In contrast, *in vivo* supplied transposase resulted in a much lower rates of remobilization in zebrafish. Transposase supplied *in vivo* using a heat shock promoter was not able to induce remobilization at early embryonic stages, but at adult stages after multiple heat shocks, Tol2 was able to remobilize at an overall rate of 6.2% [23]. . 

Thus while the injection based method is relatively labor intensive, as transposase mRNA is prepared and embryo injections performed each time remobilization is desired, the observed remobilization rate is presently higher. The breeding based strategy, while currently less efficient at inducing remobilization, is less labor intensive, only requiring setting up a mating, then collecting and analyzing resulting embryos. Thus, it would be desirable to increase the efficiency of the breeding based system to combine the benefits of both higher remobilization efficiency and reduced labor.

A number of variables have potential to be optimized to increase efficiency in the Tol2 breeding based remobilization system. Increasing the number of Tol2 donor insertions would provide more substrate transposons for remobilization. In the Sleeping Beauty breeding based study [27], a higher overall apparent remobilization rate was seen in animals from a line bearing a concatamer of 8-10 transposons (~ 1%) than in animals from a line with a concatomer of only 3 transposons (~ 0.2%). The higher rate of remobilization was partially attributed to the greater number of substrate transposons. We chose to use animals bearing one Tol2 transposon insertion in order to simplify analysis of remobilization in this study. Animals harboring multiple canonical insertions can readily be created by injection or breeding based methods to potentially increase remobilization efficiency in future studies.

More precise titration of the transposase dose necessary to induce remobilization could increase remobilization efficiency. Southern blots to determine transposase transgene copy number or quantitative PCR to determine transposase levels more precisely in our remobilized line would be beneficial in this regard. In addition, efficiency could be improved with a “next generation” transposase, similar to the “hyperactive” Sleeping Beauty transposase SB100X, which has 100 times the activity of the original SB10 form [36].

Choice of promoter is another variable that could affect efficiency of remobilization. A sperm specific promoter would be ideal for maintaining stable remobilized transgenic lines, as maternal transposase protein produced by female frogs harboring transposase contructs with an egg or ubiquitous promoter would likely cause continued remobilization in subsequent generations, preventing the stable transmission of remobilized transposons.

When developing our approach, we attempted to create a sperm specific transposase line, but this became technically challenging and unsuccessful. We created transposase animals containing somatic (Ef1α) expression constructs, but these have yet to be tested. We chose to focus on the zp3 promoter, as we were successful in generating the lines, and we reasoned that some tissue specificity might be beneficial. zp3 has been reported as an egg specific glycoprotein in a number of species including zebrafish, human and mouse [31]. We thus expected egg specific transposase expression from our construct. Our RT-PCR results indicated expression not only in the egg, but in embryos of female frogs and testis from adult male frogs (Figure 1B). –RT controls indicate the result is not due to contamination of the samples. These results suggest perhaps the zebrafish zp3 promoter is functioning less specifically in frog.

In addition to finding non-oocyte transposase expression, we also found relative differences in the amounts of transposase expressed among the three ZP3T2γGMN lines (Figure 1B). Different transposase transgene insertion loci, insertion copy numbers in the concatemer, and a variety of local host factors may result in differing expression levels among the different transposase lines, thus affecting the efficiency of transposon remobilization. Interestingly, in spite of the relatively high transposase expression observed, the U1946**♂** line produced no detectable remobilizations. It is possible that this line was affected by “overproduction inhibition”, in which transposase excess over a threshold level has been shown to inhibit the excision of transposons. This is known to occur most notably in Sleeping Beauty, but has also recently been shown for Tol2 transposase in human Hela cells [25,26]. Since our results suggest that expression levels are important for Tol2 mobilization, quantitative PCR may be useful for further optimizing remobilization. However, empiric testing is likely to remain necessary since the relationship between mobilization and expression levels is complex. Southern blot analysis to determine the number of insertions present in the concatemers of the three lines may be useful in guiding development of efficient transposase lines for future studies.

Remobilizations found on the donor chromosome, or relatively close to the donor insertion, are frequently referred to as “local hops” [23,24,27], and are desirable when attempting to saturate a genic region in gene or enhancer trapping strategies. Previous studies in frog and other species have reported local hopping of the Tol2 transposon, with multiple remobilizations less than 5 Mb from the donor insertion [22-24,37]. The injection based study of Tol2 remobilization in *X. tropicalis* reported 20% of remobilized transposons integrated near the donor locus [37]. In zebrafish, an average 14-20% local hopping frequency was found in a Tol2 remobilization study in which different donor loci were used, as well as different methods of supplying transposase (mRNA injection, single or multiple heat shocks) [23]. In contrast, 80% of Sleeping Beauty transposons were found to integrate within 3 Mb of the original insertion [27], consistent with previous reports that Sleeping Beauty tends to remobilize locally [38]. In our study, we found 60% (18) of confirmed remobilizations were on the same scaffold as the parental insertion, with 50% being within 5 Mb of the donor insertion, and 22% within 1 Mb of the donor (Figure 3A). This frequency is higher than previous rates reported for Tol2 and closer to rates reported for Sleeping Beauty.

Tol2 has also been reported to favor insertion into transcriptional units and 5’ genic areas [24-26], a highly desirable feature for enhancer and gene trapping strategies. This is in contrast to Sleeping Beauty, which has been reported to favor intergenic regions and shows little tendency to target 5’ gene regions [26]. One study of Tol2 reintegration in zebrafish has suggested an arbitrary 5 kb interval at the 3’ and 5’ ends of genes as encompassing a gene’s regulatory region [24]. We found a high percentage, 61% (11), of local remobilizations were within or less than 5 kb from a gene (Figure 3B, Table 2). While this may be partially attributed to the relatively gene rich area in which the donor transposon is located, we also found 50% (6) of remobilizations on non-donor scaffolds were in or within 5 kb of a gene (Figure 3B, Table 2). Additionally, 90% (9) of remobilizations actually within a gene were in introns in the 5’ region of the gene (Figure 3B, Table 2). Thus, our findings again suggest that breeding based strategies using Tol2 will have applications in enhancer and gene trapping studies.

Future breeding based remobilization strategies could potentially take advantage of the differences in transposon systems such as Tol2 and Sleeping Beauty with complementary or synergistic approaches to enhancer or gene trapping. For instance, a transposon construct in which a Sleeping Beauty cassette bearing the desired cargo is nested in a Tol2 construct could be used to insert and remobilize insertions to genic areas via Tol2 transposase, then the high tendency for local hopping of Sleeping Beauty could be exploited to locally saturate the area for increased enhancer or gene trapping.

## Summary

Our results demonstrate Tol2 transposon remobilization can occur in *X. tropicalis* using a breeding based strategy, and the re-integrated transposons are stably transmitted through the germline. This method could be used for enhancer trapping to create useful reporter lines. Improvements to the method, such as an increase in the number of substrate transposons, more precise titering of transposase expression, or a more efficient transposase could improve the remobilization efficiency. In the future, by selecting a transposon with relatively weak basal expression (a minimal promoter), remobilization could trap local enhancers, generating localized, specific GFP expression that may prove particularly useful for different experimental applications.

We found remobilized Tol2 transposons frequently landed near or in genes, a factor which could facilitate the trapping of genes or enhancers. Additionally, remobilizations showed a tendency to land close to the donor insertion, which further benefits gene trapping when the donor transposon is in a gene rich area, or near a gene of interest. With refinements and further work, this breeding based strategy could generate many useful transgenic lines in *X. tropicalis*, further advancing this already useful model.

## Supporting Information

Figure S1 **Generation and Analysis of Double Transgenic Lines.** Diagram showing creation of three ZP3T2γGMN transposase lines. For each line (A,B,C) we created transgenic animals U1946♂, U1984♀ and U1985♀ using the meganuclease method [33]. These animals were raised and outcrossed to create three F1 ZP3T2γGMN transposase clutches, C882, C913 and C914. We outcrossed these F1 animals to confirm a single insertion of the transposase trangene (50% GFP+^eye^ offspring). We then crossed one F1 from each transposase clutch with an Ef1αGFPTol2 animal to produce F2 double transgenic offspring. We outcrossed members of these F2 clutches and screened F3 offspring for differences in fluorescent expression and tested for remobilized transposons.
A, C. In the U1946♂ and U1985♀transposase lines, no F3 embryos with High Intensity expression were seen, and no remobilization was found in F3 embryos by LM-PCR. These lines were not tested further.
B. In the U1985♀ transposase line, a small percentage of F3 embryos showed a High Intensity expression, as well as remobilization of the substrate Ef1αGFPTol2 transposon. F3 High Intensity embryos from F2 double transgenic U2522♂ were were raised, tested by LM-PCR to confirm remobilization, and outcrossed. Germline transmission of remobilized transposons was confirmed in F4 embryos by LM-PCR.
(TIF)

Table S1 **Primers.** List of primers used in various experiments (Column 1) and their names (Column 2) as described in the Methods section of the text. Primer sequences are presented 5’ to 3’ (Column 3) with descriptions of their function (Column 4).
^1^. “Biotin-L” is abbreviation of “Biotinylated primer to Tol2 5’ arm sequence” from [35].
(DOC)
