# Supplementary figures and images for: Breeding Based Remobilization of Tol2 Transposon in Xenopus tropicalis
Source: PLoS One. 2013 Oct 8;8(10):e76807. doi: 10.1371/journal.pone.0076807 (PMC3792888; doi:10.1371/journal.pone.0076807)

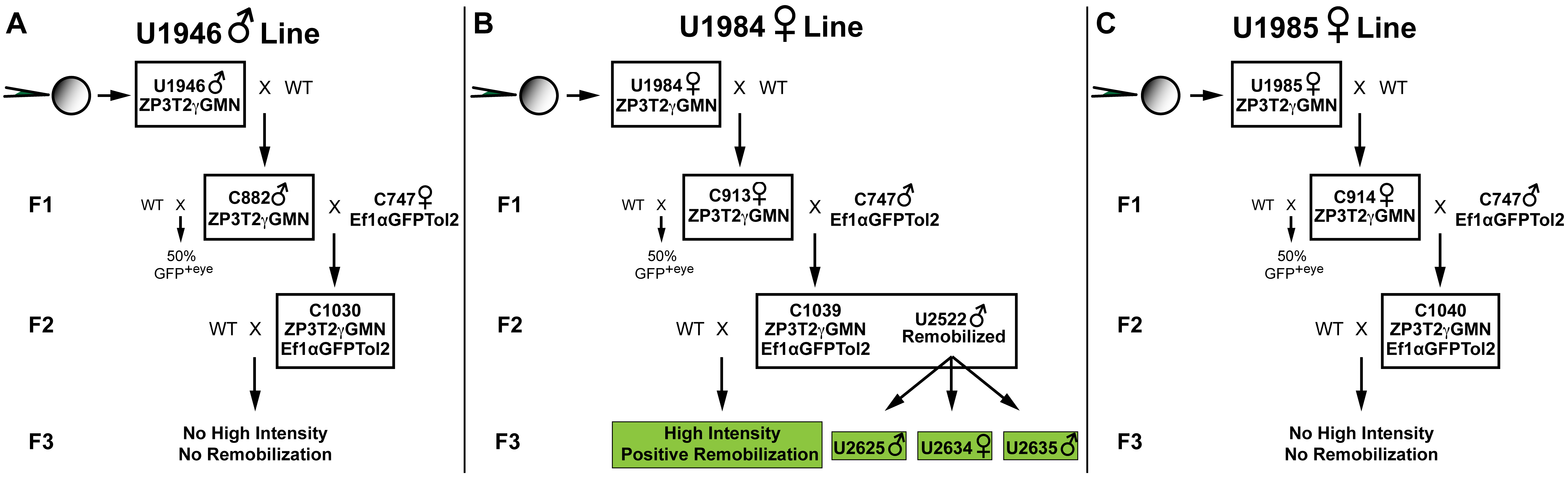

Supplement: Figure S1 — Generation and Analysis of Double Transgenic Lines. Diagram showing creation of three ZP3T2γGMN transposase lines. For each line (A,B,C) we created transgenic animals U1946♂, U1984♀ and U1985♀ using the meganuclease method [33]. These animals were raised and outcrossed to create three F1 ZP3T2γGMN transposase clutches, C882, C913 and C914. We outcrossed these F1 animals to confirm a single insertion of the transposase trangene (50% GFP+eye offspring). We then crossed one F1 from each transposase clutch with an Ef1αGFPTol2 animal to produce F2 double transgenic offspring. We outcrossed members of these F2 clutches and screened F3 offspring for differences in fluorescent expression and tested for remobilized transposons. A, C. In the U1946♂ and U1985♀transposase lines, no F3 embryos with High Intensity expression were seen, and no remobilization was found in F3 embryos by LM-PCR. These lines were not tested further. B. In the U1985♀ transposase line, a small percentage of F3 embryos showed a High Intensity expression, as well as remobilization of the substrate Ef1αGFPTol2 transposon. F3 High Intensity embryos from F2 double transgenic U2522♂ were were raised, tested by LM-PCR to confirm remobilization, and outcrossed. Germline transmission of remobilized transposons was confirmed in F4 embryos by LM-PCR. (TIF) [file pone.0076807.s001.tif]
