# Supplementary material for: Breeding Based Remobilization of Tol2 Transposon in Xenopus tropicalis
Source: PLoS One. 2013 Oct 8;8(10):e76807. doi: 10.1371/journal.pone.0076807 (PMC3792888; doi:10.1371/journal.pone.0076807)
Supplement: Table S1 — Primers. List of primers used in various experiments (Column 1) and their names (Column 2) as described in the Methods section of the text. Primer sequences are presented 5’ to 3’ (Column 3) with descriptions of their function (Column 4). 1. “Biotin-L” is abbreviation of “Biotinylated primer to Tol2 5’ arm sequence” from [35]. (DOC) [file pone.0076807.s002.doc]

|  | **Primer** | **Primer sequence (5’ – 3’)** | **Comment** |
| --- | --- | --- | --- |
| **RT-PCR** | tol2_transposase_F | TGCAAAGAAGCAGGAGAAGG | Forward PCR primer |
|  | tol2_transposase_R | TTCCTTGTTGTAGGGCATCC | Reverse PCR primer |
|  | odcI_exon6_F | TGTTCTGCGCATAGCAACTG | RT-PCR control primer |
|  | odcI_exon6_R | ACATCGTGCATCTGAGACAGC | RT-PCR control primer |
| **LM-PCR** | Biotin-L^1^ | BIO-AAACTGGGCATCAGCGCAATTCAAT | Primer extension 5’ transposon arm^35^ |
|  | Tol2 5’ N1 | CGCAATTCAATTGGTTTGG | Primary PCR primer: 5’ transposon arm^35^ |
|  | Tol2 5’ N2 | GCAAGGGAAAATAGAATGAAG | Nested PCR primer: 5’ transposon arm^35^ |
|  | Biotin-R | BIO-ACCAGGGAGTCTCTGCTCAC | Primer extension 3’ transposon arm |
|  | N1-R | CCTGCTATTTGCAGCCTCTC | Primary PCR primer: 3’ transposon arm |
|  | N2-R | CAGCCCCAAAAGAGCTAGG | Nested PCR primer: 3’ transposon arm |
|  | Seq1-R | GCGTGTACTGGCATTAGATTG | Sequencing primer: 3’ transposon arm |
|  | Seq2-R | AAACCTTGTATGCATTTCATTT | Sequencing primer: 3’ transposon arm |
|  | NZ117 | GACCCGGGAGATCTGAATTCAGTGGCACAGCAGTTAGG | Linker primer^35^ |
|  | NZ118-P | pCCTAACTGCTGTGCCACTGAATTCAGATCTCCC | 5’ phosphorylated linker primer^35^ |
|  | OCI | GACCCGGGAGATCTGAATTC | Primary PCR primer specific to linker^35^ |
|  | OCII | AGTGGCACAGCAGTTAGG | Nested PCR primer specific to linker^35^ |
| **Genomic** | OI_L | GCCAGACCTTTGTGAGAAGC |  |
|  | OI_R | GCCTCCTGTGCTATAAGTGG |  |
|  | U2522_1_L | AGTGTCAGCTCCTTCACAGG |  |
|  | U2522_1_R | GAGAGGCCTCAGGGAGAGAC |  |
|  | U2522_2_L | GCCTTTGTTCTTCCTTCAGC |  |
|  | U2521_1_L | CAGCACAGACATGGTTCTCC |  |
|  | U2521_1_R | GGGCTCATGGAAAGTAGAAG |  |
|  | F#5_1_L | TTAGCAGCACTAGCAGAATGC |  |
|  | F#5_1_R | GAAGCTGCACTCATGTAGCC |  |
|  | F#5_2_R | CCCATACTGTGAGCAAGCAG |  |
|  | F#5_3_L#1 | CAAGCATTCACGGTCAGTTG |  |
|  | F#5_3_L#2 | GGCATCAGTATTGTCAGTACCC |  |
|  | F#5_3_R | AGCTGTATGCATTCTACTCTGC |  |
|  | F#6_1_L | TATCAGAGAGCCATGCAAGC |  |
|  | F#6_1_R | TATCATTGACAGCGCAGAGC |  |
|  | F#6_2_L | GCCAGGCTAACATAACTGTTCC |  |
|  | F#6_2_R | CCATCTTGACTGGGATGTGG |  |
|  | F#6_3_L | CATTGAGCGGGAAGTTGC |  |
|  | F#6_3_R | CAACAACAAGTTCCAGTGACC |  |
|  | HI #5_1_R | CACTTACAAGGGCCATAGTGC |  |
|  | HI #5_1_L | CGACTGCTTTCAGGTCAAGC |  |
|  | HI #6_1_R | TGAAACACTCCAACCTTTACCC |  |
|  | HI #6_1_L | GCAAATAACTCAGCCCATATACCC |  |
|  | HI #7_1_R | GCTCTTCTTCTCCCATGACG |  |
|  | HI #7_1_L | GACCAAATTAAGGGCCTTGC |  |
|  | HI #8_1_R | AGGGAGGCACTTATTGATGG |  |
|  | HI #8_1_L | CACCATAGGTGGATGGAAGC |  |
|  | HI #8_2_R | GTGCTCTCTGTTGCAGTTGG |  |
|  | HI #8_2_L | TTCCGATCCGTGTTCTTACG |  |
|  | HI #10_1_R | TTCGGCATAAACTCACAAGC |  |
|  | HI #10_1_L | GACCTTTCTCTGCCTGTTGC |  |
|  | HI #12_1_R | GTGGGACAGATAACGCAAGG |  |
|  | HI #12_1_L | TCAGTCTGCATCCTTGTTGC |  |
|  | HI #14_1_R | ACGCACGCCATGTTAGCC |  |
|  | HI #14_1_L | GCAGACACCCAGTCTTTGG |  |
|  | HI #17_1_R | CACAACAGCTGGAGAGTTGC |  |
|  | HI #17_1_L | CCAGGACTGAGGTAGATGAGC |  |

**Table S1. Primers**
